# The Onto-Rhythmic Self: An Ontological Reframing of Subjectivity

**DOI:** 10.1007/s12124-025-09937-0

**Published:** 2025-10-01

**Authors:** Mohammad Dawood Rahimi

**Affiliations:** Freie Universität Berlin, Berlin, Germany

**Keywords:** Selfhood, Ontology, Consciousness, Time, Husserl, Dennett, Rhythmic modulation, Phenomenology, Expressive being, Participatory epistemology

## Abstract

In the present work of intellect, we introduce a novel approach to selfhood, the Onto-Rhythmic Self, situated within a participatory ontology in which Consciousness and Time function not as substrates but as expressive modalities of Being. Through a comparative critique of Husserl’s transcendental ego and Dennett’s narrative self, we classify four axes of limitation: the Who of subjectivity, the How of temporal articulation, the What of ontological grounding, and the Why of existential necessity. To address such lacunae, we use a multi-layered method combining comparative ontological analysis, phenomenological reflection, and expressive mapping. Such a method discloses the limitations of synthesis (Husserl) and simulation (Dennett) and instead articulate the self as a modulatory field of Being—a dynamic site where rhythm, resonance, and relational articulation converge. The outcome features that the Onto-Rhythmic Self is neither a transcendental subject nor a computational fiction, but an ontological inscription that discloses across neural, cultural, ecological, and quantum strata. Its implications extend across domains: philosophically, it reframes individuation as ontic emergence; clinically, it reorients therapy toward rhythmic re-attunement; and artistically, it reconceives creation as ontological modulation rather than symbolic representation. By restoring depth, relational presence, and semantic coherence to the discourse on selfhood, through the Onto-Rhythmic Self, we add a structurally rigorous and experientially faithful grammar of consciousness, which is a new mode of listening to the rhythm of Being.

## Introduction

The concept of the self has long occupied an argumentative position in philosophy, psychology, and cognitive science. From Descartes’ cogito—the self as the indubitable ground of knowledge (Meditations, 1641/2008 (Descartes, 2008)—to Hume’s bundle theory of perceptions (A Treatise of Human Nature, 1739/2000, I.iv.vi (Hume, 2000), the Western tradition has lingered between substantialist and eliminativist accounts of selfhood. In the German idealist history, the self is featured as the condition for the possibility of experience (Kant, Critique of Pure Reason, 1781/1908, B131–B134 (Kant, 1908), while in phenomenology, the position is radicalized into the transcendental ego, described as the constitutive pole of intentionality (Husserl, Ideas I, 1913/1989, 46–49 (Husserl, 1989). Meanwhile, within the analytic tradition, the self is often reduced to questions of identity, memory, or functional continuity (Parfit, Reasons and Persons, (Parfit, 1987), Part III, pp. 418–431 (Dennett & Dennett, 1993).

Among these thinkers, Husserl’s (1989) transcendental ego first posits a constituting consciousness that synthesizes temporality and experience through retention, protention, and primal impression (Ideas I, 79–83). For Husserl, the ego is not an empirical entity but rather “the pole of intentional acts, the center from which all meaning radiates” (Ideas II, p. 57). In contrast, Dennett (1993), where arguing within a functionalist and computational framework, introduces the self as a “center of narrative gravity,” a theorist’s fiction generated by recursive linguistic and cognitive mechanisms (pp. 418–431).

Despite their radically different methods and metaphysical premises, they converge on a shared presupposition: that the self is either a structuring subject (Husserl) or a computational byproduct (Dennett). Correspondingly, each inherits the Cartesian bifurcation between substantial subjectivity and mechanistic objectivity, albeit in transformed registers. The result is a neglect of the ontological density of lived experience, the rhythmic and affective textures of temporality, and the relational resonances through which selfhood is enacted.

The limitations of their approaches to self can be organized along four critical axes:

- **Who**: Husserl pivots on a disembodied constituting subject (Ideas I, 80–81), while Dennett insists on a mechanistic processor (Consciousness Explained, p. 431). Together, they occlude the embodied, ecological, and relational scopes of selfhood (cf. Zahavi, Subjectivity and Selfhood, 2005, pp. 13–29 (Zahavi, 2005).
- **How**: The temporal synthesis in Husserl and the narrative construction in Dennett both rely on representational schemas, thereby neglecting non-linear, rhythmic, and resonant modalities of disclosure (cf. Merleau-Ponty, Phenomenology of Perception, 1945/2012, pp. 137–152 (Merleau-Ponty, 1945; Merleau-Ponty, 2012).
- **What**: Neither account incorporates semantic resonance and expressive modulation as ontological categories, dimensions accentuated in contemporary ontology (Deleuze, Difference and Repetition, 1968/1994, pp. 70–93 (Deleuze, 1968, 1994).
- **Why**: By situating consciousness as either transcendental or computational, both models miss its participatory and expressive nature—its inscription within Being itself (cf. Heidegger, Being and Time, 1927, 9 (Heidegger, 1927).

To situate such critiques historically, one must traverse the conceptual terrain from classical metaphysics (where selfhood was bound to soul, substance, or rational essence), through phenomenology and existentialism (where it was reconceived in terms of lived embodiment and relationality), to cognitive science and post-structuralism (where the self is modeled as simulation, narrative, or de-centered function).

Such a trajectory divulges the limitations of synthesis (Husserl) and simulation (Dennett) and simultaneously prepares the ground for an alternative approach: the Onto-Rhythmic Self—a model of selfhood that functions as a modulatory field, constituted through resonance, articulated in semantic rhythm, and attuned ontologically to the unfolding of Being.

## Analytical Foundations: Four Axes of Conceptual Limitation

Despite their enduring influence, both Husserl’s transcendental ego (Ideas I, 1913) and Dennett’s narrative self (1993) exhibit foundational limitations when explained through the approach of ontological articulation. While their models have shaped dominant discourses on subjectivity, they are still anchored in paradigms that obscure the participatory, expressive, and ontologically grounded nature of consciousness. Each reduces the self either to a disembodied synthesizer of intentional acts (Husserl, Ideas I, 46–49) or to a computational processor of narrative threads (Dennett, Consciousness Explained, pp. 418–431), abstracting lived experience into representational or mechanistic frameworks.

To advance a philosophy in which Consciousness and Time are not substrates but modalities of Being, we must interrogate the conceptual scaffolding underwriting such views. Classical and contemporary frameworks—from Husserl’s idealist synthesis to Dennett’s functionalist abstraction—fail to capture the semantic density, relational attunement, and ontic texture of selfhood. What is required is not revision internal to such paradigms, but a departure from them: a grammar of Being that foregrounds resonance, modulation, and expressive articulation.

To prepare the ground for such a reframing, we explain and examine four conceptual axes—Who, How, What, and Why—each exposing a structural misalignment between inherited models and the lived texture of consciousness.

### Who: The Misframing of the Self as Subject or Processor

Both Husserl and Dennett misidentify the “who” of selfhood, reducing it to a transcendental subject (Husserl) or a computational fiction (Dennett). For Husserl, grief, for instance, is parsed as a series of intentional acts directed toward the memory of the deceased, temporally synthesized through retention and protention (Ideas I, 77–83). The grieving self is construed as a transcendental ego—a constituting pole abstracted from lived entanglement. The ontological rupture of grief, its relational wound, disappears into a schema of intentional synthesis. Nonetheless, for Dennett, grief registers as a disruption in the autobiographical narrative generated by the brain. The self is no more than a “center of narrative gravity,” a functional construct that reconfigures stories to accommodate absence (Consciousness Explained, pp. 418–431). The existential gravitas of grief, the reshaping of Being itself, is flattened into narrative repair.

#### Our Critique

Their accounts misframe the “who” by construing the self as either a detached subject or a computational artifact. In our grammar, the grieving self is a modulatory field—attuned to absence, reshaped by loss, rhythmically inscribed by Being itself.

## How: The Reduction of Consciousness to Representation and Sequence

By imposing sequential construction upon emergent phenomena, consciousness is reduced to either intentional representation (Husserl) or recursive narrativization (Dennett). For example, in improvisational music, Husserl would describe consciousness as the temporal synthesis of retention, impression, and protention (Lectures on Internal Time-Consciousness, 10–12 (Husserl, 2012b). Such a view frames improvisation as a structured succession of acts, subordinating its fluidity to linear temporality.

On the other hand, Dennett (1993) interprets improvisation as a story the brain tells itself, a recursive narrative update within the system’s cognitive economy (Consciousness Explained, pp. 418–425). Improvisation becomes representational bookkeeping rather than ontological emergence.

### Our Critique

They presume that consciousness is assembled through representational sequence. In our grammar, improvisation is not narrativized construction but ontic articulation: a direct modulation of Being, enacted in real time.

## What: The Erasure of Semantic Resonance and Ontic Texture

By translating it into representational or computational terms, they obscure the expressive resonance of lived meaning. For instance, for Husserl, a line of poetry that moves one to tears is an intentional relation between noesis and noema (Ideas I, 84–87). The affective rupture is subordinated to the formal structure of meaning-bestowal, neglecting the ontological tremor that transforms the speaker’s Being. That is, for Dennett, the same moment is reduced to narrative integration, a computational adjustment in autobiographical self-modeling (Consciousness Explained, pp. 418–420). The tear is coded as information rather than ontological resonance.

### Our Critique

Although they erase the ontic pulse of meaning, in our grammar the tear is not a reaction but a semantic gesture: an inscription of Being through expressive modulation.

## Why: The Paradigmatic Misalignment with Ontological Grounding

In the same vein, Husserl and Dennett situate consciousness within paradigms that either transcend the world (Husserl) or reduce it to computational mechanism (Dennett), thereby occluding its participatory grounding. Consider, for instance, the case of language acquisition. For Husserl, language arises through the constitutive acts of the ego, which confer sense upon signs (Ideas I, §§ 124–128). Yet such an idealist account abstracts language from embodied presence and relational immersion. By contrast, Dennett interprets language learning as the continual updating of a narrative self-model—symbols decoded and integrated into cognitive routines (Consciousness Explained, pp. 416–423). Here language is reduced to algorithmic processing, stripped of its ontological rooting in shared Being.

### Our critique

Both thinkers misplace consciousness—Husserl elevating it above the world, Dennett collapsing it beneath. In our grammar, consciousness is neither transcendent nor reductive but immanent: a participatory field in which Being articulates itself through resonance and relation. A child’s speech is not synthesized as data nor constituted as mere ideality; it is ontologically inscribed, arising from the depth of shared presence.

## Conclusion: Fourfold Critique

Structured around Who, How, What, and Why, our fourfold critique does not solely disclose gaps in Husserl and Dennett; it divulges a structural foreclosure. Their accounts of selfhood are still bound by representational and functionalist assumptions that prevent genuine engagement with the ontological density of the entity. In consequence, the self is persistently mischaracterized as either observer or processor, whereas phenomenological disclosure shows it as a modulatory field of Being in which rhythm, resonance, and articulation converge.

In contrast, we contend that the self is not a product of representation or computation but an ontological inscription—not a synthesis of acts but a gesture of presence. Our reconfiguration establishes the conceptual terrain for the hypotheses of the Onto-Rhythmic Self, a model of consciousness that aims to be simultaneously structurally rigorous and experientially faithful.

### Hypotheses

Having established the conceptual limitations of classical and contemporary models of selfhood, particularly their misalignment with the expressive and ontological nature of consciousness, we now articulate a set of hypotheses that formalize the departure from representational and computational paradigms. These hypotheses are not solely theoretical propositions; they are ontological commitments. They aim to reframe the self not as a subject or a processor, but as a modulatory field of Being, enacted through resonance, articulation, and temporal depth. Each arises directly from the fourfold critique outlined above—Who, How, What, and Why—and gestures toward a new grammar of consciousness that is structurally rigorous, historically situated, and experientially faithful.

- **H1**: Classical and contemporary models of selfhood—exemplified by Husserl’s transcendental ego (Husserl, Ideas I, 46–49) and Dennett’s narrative self (Dennett, Consciousness Explained, p. 418)—are insufficient for explaining the ontological depth, relational attunement, and expressive coherence of consciousness.
- **H2**: The self is not a representational or narrative construct, but a semantic field of ontic modulation—a site where meaning is not processed but enacted, not stored but inscribed (cf. Derrida, Speech and Phenomena, 1973, pp. 140–141 (Derrida, 1973); Foucault, Hermeneutics of the Subject, 1982–83/2005, pp. 369–372 (Gros, 2005).
- **H3**: Consciousness and Time are not substrates or containers of experience, but expressive dimensions of Being; the self emerges through their rhythmic articulation, not through synthetic constitution (Husserl) or computational simulation (Dennett).

### Testing the Hypotheses

The methodological task is not empirical in the positivist sense, but phenomenologically grounded and philosophically integrative. Our aim is not falsification but ontological elucidation and explanation: to disclose where inherited models falter and where a new grammar of Being begins to resonate. We approach such a testing phase through three axes:

- **Ontological Assumptions**: How is the self-grounded—essence, function, or field?
- **Temporal Structure**: How is time conceived—linear, synthetic, narrative, or rhythmic?
- **Epistemic Function**: How does the self-know—through representation, narration, or attunement?

This mode of inquiry aligns with the genealogical trajectory traced from classical phenomenology (Husserl, 2012a), through analytic functionalism (Dennett & Dennett, 1993), into post-structuralist critiques of subjectivation and différance (Derrida, 1973; Gros, 2005). It thereby situates the Onto-Rhythmic Self as a constructive response to both traditions.

#### Comparative Frameworks of Selfhood: Ontology, Temporality, Knowing

To situate the Onto-Rhythmic Self within the ongoing discourse on subjectivity, it is essential to recognize the divergent ontological assumptions that have shaped the modern understanding of selfhood. Philosophical accounts from Husserl to Dennett have framed the self either as a transcendental condition of possibility or as a computationally generated fiction, each offering coherence but also significant limitations. By contrast, the Onto-Rhythmic Self advances a participatory ontology in which selfhood emerges as rhythmic modulation rather than as transcendental substance or narrative construct. Such a comparative positioning is summarized in Table 1: Comparative Frameworks of Selfhood—Ontology, Temporality, Knowing, which contrasts the ontological grounding of Husserl (1913), Dennett (1993), and the present model (see Table 1).

**Table 1:** Ontological grounding: What is the self?

Model	Ontological Assumption	Nature of Self
Husserl (1913)	Transcendental idealism (*Ideas I*, 46–49 (Husserl, 1989))	The self is a pure, constituting ego—*transcendental subjectivity*—which gives intentional form to experience. Not empirical but the condition of possibility.
Dennett 1993	Functionalist materialism (Consciousness Explained (Dennett & Dennett, 1993), p. 418)	The self is a “center of narrative gravity,” a useful fiction generated by cognitive processes.
Onto-Rhythmic Self	Participatory ontology; cf. (Thompson, 2010; Varela Francisco et al., 1991)	The self is a modulatory field of Being, emergent through relational resonance and ontic articulation, not computation or transcendence.

Summary of ontological assumptions and models of selfhood in Husserl, Dennett, and the Onto-Rhythmic Self.

While Husserl offers a structurally coherent transcendental foundation, it abstracts from embodied and relational life (Zahavi, 2005). Dennett, conversely, trivializes the self into a computational narrative, lacking existential depth (Metzinger, 2004). Our model rejects both extremes, grounding the self as ontologically enacted modulation rather than subject or fiction.

The persistence of selfhood has long been interpreted through contrasting models of temporality: Husserl’s synthesis of retention and protention, Dennett’s narratively revised autobiography, and our proposal of rhythmic enactment. As outlined in Table 2: Temporal Structure—How Does the Self Persist? —such a shift from linear construction to rhythmic resonance redefines continuity not as cognitive fabrication but as ontological coherence (see Table 2).

**Table 2:** Temporal structure: How does the self persist?

Model	Time and Continuity	Implication
Husserl (1913)	Time synthesized through retention, impression, protention (*Lectures on the Phenomenology of Internal Time-Consciousness*, 18–20 (Husserl et al., 1994)).	The ego as architect of temporal continuity.
Dennett (1993)	Time as narrative construction—revised autobiographical story (p. 418).	The self as a fragile, revisable fiction.
Onto-Rhythmic Self	Time as rhythmic enactment—emergent pulses, not linear synthesis.	Continuity grounded in resonance and coherence, not narrative or synthesis.

Comparative models of temporal continuity in Husserl, Dennett, and the Onto-Rhythmic Self.

Such a shift from linearity to rhythm aligns with contemporary findings in trauma studies and music therapy, where rhythm fosters temporal reintegration (Faulkner, 2017; McFerran et al., 2020). Rhythm here is not metaphorical but ontological.

The epistemic dimension of selfhood concerns not only what the self is or how it endures, but how it comes to know. Table 3: Epistemic Function—How Does the Self Know? contrasts Husserl’s eidetic reduction, Dennett’s narrative modeling, and our proposed account of participatory attunement (see Table 3).

**Table 3:** Epistemic function: How does the self know?

Model	Mode of Knowing	Limits
Husserl (1913)	Phenomenological reduction to essences.	Risks solipsism; detaches from relational presence.
Dennett (1993)	Narrative/representational modeling.	Risks trivialization; reduces self to illusion.
Onto-Rythmic Self	Attunement, resonance, participatory enactment.	Avoids both detachment and trivialization.

Contrasting epistemic modes of selfhood in Husserl, Dennett, and the Onto-Rhythmic Self.

Our reframing of knowing as ontological intimacy resonates with enactive cognition (Noë, 2004; Varela Francisco et al., 1991) and post-structuralist accounts of différance (Derrida, 1973), where meaning is enacted relationally rather than fixed representationally.

#### Toward the Onto-Rhythmic Self

The comparative framework confirms that Husserl and Dennett provide influential but structurally incomplete accounts of selfhood. The Onto-Rhythmic Self arises as a philosophical necessity, offering:

- Ontological depth (field, not subject or fiction),
- Rhythmic temporality (resonance, not synthesis or story),
- Participatory epistemics (attunement, not representation).

Our paradigm restores presence, depth, and semantic resonance to discourses on consciousness, bridging phenomenology, analytic philosophy, and post-structuralism within a coherent grammar of Being.

###  Historical Trajectories of Selfhood: from Essence to Modulation

#### Classical Foundations: Soul, Substance, and Rational Principle

In antiquity, the self was construed as a metaphysical essence—immortal, rational, and hierarchically ordered. Plato (c. 380 BCE) framed the self through his tripartite soul—reason, spirit, and appetite—arguing in the Republic that justice arises when each part fulfills its function (436a–441c; cf. (Annas, 2009). The soul thus appeared as a transcendent principle oriented towards eternal Forms. Aristotle, by contrast, defined the soul (psyche) not as a separable substance but as the form of the living body, the actuality of its potential (De Anima II.1, 412a–413a; (Taylor, 2009).

Early Christian thinkers transformed these legacies. Augustine (397 CE), in Confessions XI, conceived the self through memoria and interior reflection, locating truth in divine illumination (XI.20, p. 264; (Clark, 1993). Plotinus (c. 270 CE) articulated a hierarchical ontology in which the soul ascends from multiplicity toward the One, layering selfhood through emanations (Enneads VI.9.8–11; (Armstrong, 1966).

Despite their metaphysical richness, their accounts are largely static: the self as essence, teleologically framed, insufficiently attentive to temporality, embodiment, and modulation. Selfhood here was more substance than enactment.

#### Modern Thinkers: Subjectivity, Memory, and Skepticism

The modern period introduced a decisive epistemic turn. (1641/1996) grounded the self in the cogito— “I think, therefore I am”—establishing subjectivity as indubitable foundation (Meditations II, p. 19; Descartes (Descartes, 1709). Locke (1690/1847) departed from substance, locating personal identity in memory and psychological continuity (Essay, II.xxvii.9–16). Hume (1739/2000) radicalized this critique, denying any stable self and reducing it to a “bundle of perceptions” (Treatise, I.iv.vi, p. 252; (Hume, 2000). Kant (1781/1998), rejecting metaphysical substance, sought to restore unity through the transcendental apperception—the “I think” that must accompany all representations (Critique of Pure Reason, B132–B134).

Their models advanced conceptual rigor but rendered the self increasingly abstract—either as rational substrate (Descartes, Kant), as psychological continuity (Locke), or as skeptical residue (Hume). Ontological and expressive depth gave way to cognitive scaffolding.

#### Phenomenological and Existential Thinkers: Embodiment and Ontological Openness

The twentieth century reoriented selfhood toward embodiment and existential situatedness. Husserl (1913/1989) defined the transcendental ego as the constituting pole of intentionality, synthesizing temporality through retention, protention, and primal impression (Ideas I, 79–83; Ideas II, p. 57; (Husserl, 1989). As Heidegger (1927/1962) argues in Being and Time, selfhood is reframed as Dasein, an ontological openness grounded in being-in-the-world (pp. 67–83; (Heidegger, 1927). Sartre (1943/2022) radicalized this into freedom-as-negation, where the self is a project perpetually escaping itself (Being and Nothingness, pp. 59–73; (Sartre et al., 2022). Merleau-Ponty (1945/2012) placed embodiment at the center, describing the body as “our general medium for having a world” (Phenomenology of Perception, p. 169).

These thinkers moved decisively beyond static substance models, situating the self in live temporality, embodiment, and intersubjectivity. Yet, their frameworks often retained linear or representational assumptions about time and synthesis, limiting their grasp of resonance and rhythmic modulation.

#### Contemporary Thinkers: Narrative, Emergence, and Illusion

Emerging from neuroscience, analytic philosophy, phenomenology, and post-structuralism, contemporary accounts treat the self as emergent, narrative, or illusory. Dennett (1991) proposed the self as a “center of narrative gravity,” a theorist’s fiction generated by recursive linguistic and cognitive mechanisms (Consciousness Explained, pp. 418–431). Metzinger (2003/2004) radicalized this eliminativism, claiming “no one is behind the scenes”: only a dynamic phenomenal self-model (Being No One, pp. 1–3, 546). Damasio (1999) distinguished proto-self, core self, and autobiographical self as layered neurobiological scaffolds (The Feeling of What Happens, pp. 153–175). Zahavi (2005), resisting eliminativism, articulated a phenomenological minimal selfhood—the pre-reflective “for-me-ness” intrinsic to experience (Subjectivity and Selfhood, pp. 13–29; (Zahavi, 2005).

Post-structuralist critiques dismantled both transcendental subjectivity and computational models. Derrida (1997/1973), in Speech and Phenomena, argued that temporal synthesis presupposes différance: a constitutive deferral whereby presence is fractured by absence (pp. 77–85). Foucault (1981–82/2005), in Hermeneutics of the Subject, reframed the self as an effect of subjectivation—historically contingent practices of self-formation and discipline (Lectures 2–5, pp. 25–67; (Gros, 2005).

Together, these accounts destabilize both Husserl’s transcendental ego and Dennett’s narrative construct, showing that selfhood is neither timeless subjectivity nor computational artifact, but a differential play of traces (Derrida) and a historically embedded process (Foucault).

#### Contemporary Thinkers: Narrative, Emergence, and Illusion

Placed side by side, Husserl and Dennett are the two dominant poles of modern theorizing. Husserl’s transcendental ego grounds subjectivity in intentional synthesis, structuring temporality through retention, protention, and primal impression (Husserl et al., 1994). This account secures unity of meaning but risks disembodiment and abstraction, with the ego drifting toward solipsism (Zahavi, 2005, p.118).

Dennett, by contrast, strips away transcendental grounding, positing the self as a narratively useful fiction. The “center of narrative gravity” explains coherence across distributed processes but at the cost of ontological depth (Consciousness Explained, pp. 418–431). As critics note, such a model trivializes lived experience, rendering subjectivity narratively functional but existentially vacuous (Thompson, 2010, p.19).

Taken together, Husserl and Dennett delineate the limits of synthesis and simulation: one over-transcendentalizes, the other over-reduces. They are bound to explanatory logic that construe the self either as a constituting subject or as a computational fiction.

#### Contemporary Thinkers: Narrative, Emergence, and Illusion

The Onto-Rhythmic Self emerges not as a wholesale rejection of prior models but as a resonance with their insights and a correction of their limits. Each historical trajectory—classical essence, modern subjectivity, phenomenological constitution, computational narrative—grasps a dimension of selfhood but narrows it through static substance, linear synthesis, or functional reduction. The dialogical task is to preserve what their models feature while reframing their assumptions within a broader onto-rhythmic field.

##### Against Husserl: From Synthesis to Resonance

Husserl’s (1913) account of internal time-consciousness, structured through retention, protention, and primal impression, rightly captures the temporal constitution of experience. Yet his model presupposes a linear synthesis and a transcendental ego that gathers meaning into coherence. Through the Onto-Rhythmic perspective, we acknowledge Husserl’s insight into temporality but shifts from synthesis to resonance: time is not linearly woven but rhythmically enacted, emerging as oscillation, modulation, and phase-shift. Rather than a solitary ego that constitutes meaning, the self is a participatory field attuned to ontological rhythms.

##### Against Dennett: From Narrative Simulation to Ontological Depth

Dennett’s (1993) “center of narrative gravity” clarifies how coherence arises in cognition, but at the price of stripping subjectivity of existential gravitas. Through the Onto-Rhythmic Self, we agree that narrative contributes to the articulation of identity yet insist that narration is not sufficient. Beneath and beyond stories lies a vibratory pulse of Being—an expressive ontology irreducible to linguistic or computational scaffolding. Where Dennett sees fiction, the onto-rhythmic account discerns modulation: not a mere construct, but a field of resonance in which narration is one among many rhythms.

##### Dialoguing with Classical and Existential Legacies

Classical models rightly intuited the self as oriented beyond the empirical—toward the Forms (Plato; (Annas, 2009), the divine (Augustine; (Clark, 1993), or the One (Plotinus; (Armstrong, 1966), but froze such an orientation into essence. The onto-rhythmic account honors their intuition of transcendence but relocates it as immanently rhythmic rather than metaphysically static. Likewise, existential, and phenomenological accounts (Heidegger, Sartre, Merleau-Ponty) foreground embodiment and freedom, yet often within linear or representational frames. We, through onto-rhythm, reframe embodiment not solely as a “medium of worldhood” but as a rhythmic articulation of Being itself.

##### Toward Modulation as Ontological Grammar

In dialogue with such traditions, the Onto-Rhythmic Self proposes a shift in grammar:

- Not substance (Plato, Aristotle, Augustine), but modulation.
- Not synthesis (Husserl), but resonance.
- Not narrative (Dennett), but rhythm.
- Not pure negation or projection (Sartre), but ontological attunement.

Therefore, the self is neither an essence to be discovered nor a fiction to be discarded, but a rhythmic field of Being in which consciousness participates. Modulation becomes the grammar through which identity, temporality, and meaning articulate themselves—an ongoing play of alignment and dissonance, presence and deferral, coherence, and excess.

### Methods: Ontological Inquiry and Expressive Analysis

The methodological framework we adopted here does not seek empirical verification in the conventional sense. Instead, it is oriented toward existential grounding, conceptual rigor, and expressive adequacy. Our approach integrates comparative ontology, phenomenological reflection, expressive mapping, and ontic resonance evaluation. Each stage functions not as a technical procedure but as a mode of ontological inquiry.

#### Comparative Ontological Analysis

The first methodological moment consists of juxtaposing metaphysical commitments across Husserl, Dennett, and the Onto-Rhythmic Self. Husserl’s transcendental phenomenology places emphasis on synthesis, wherein the ego constitutes the unity of experience from a transcendental standpoint (Ideas I, 1913, 46–49). While this model rigorously analyzes intentionality, it neglects the embodied and intersubjective strata of subjectivity.

Dennett’s model, by contrast, rejects transcendental grounding and proposes the self as a “center of narrative gravity” (Consciousness Explained, 1991, p.418). This functionalist materialism captures certain explanatory features within cognitive science, yet it collapses ontological depth into representational simulation (Thompson, 2010; Mind in Life, p.19).

Against both poles, the Onto-Rhythmic Self emphasizes modulation, resonance, and semantic articulation as ontological primitives rather than derivative features. In this sense, it aligns more closely with process ontologies (Process and Reality, p.207; (Whitehead, 1929) and differential ontologies (Difference and Repetition, p.28; (Deleuze, 1968), which privilege becoming and relation over fixed identity.

#### Phenomenological Reflection

The second methodological layer involves phenomenological reflection on lived experience. Experiences such as grief, improvisation, poetic resonance, and language emergence are treated not as data points but as ontological disclosures. These events explain how selfhood modulates affective, temporal, and relational dimensions.

Here we draw on Merleau-Ponty’s claim that embodiment is the “general medium for having a world” (Phenomenology of Perception, 1945, p.146), alongside Heidegger’s account of Being-in-the-world as constitutively temporal (Being and Time, 1927, 65–68). Such reflections feature that resonance is not metaphorical but a constitutive principle of subjectivity, whereby the self emerges through rhythms of affective and temporal attunement.

#### Expressive Mapping

The third methodological moment, expressive mapping, seeks to trace articulations of selfhood across multiple ontological strata: neural rhythms, cultural practices, environmental attunements, and quantum amplitudes. Such an approach is inspired by enactivist and neurophenomenological accounts, which signify that consciousness cannot be reduced to computation but is enacted through embodied and embedded dynamics (The Embodied Mind, p.149; (Varela Francisco et al., 1991).

Unlike symbolic or representational mapping, expressive mapping is ontological. It identifies the semantic fields through which the self becomes legible as a modulatory field of Being. Such a perspective resonates with Jan Zwicky’s emphasis on intermodal coherence in philosophy, where meaning arises through resonant alignment across heterogeneous domains (Lyric Philosophy, p.47; (Zwicky, 1992).

#### Ontic Resonance Evaluation

Finally, the adequacy of ontological models is assessed through ontic resonance evaluation. Unlike empirical validation, which relies on measurement and prediction, this criterion evaluates a model’s coherence through existential alignment and semantic depth. The central questions are: Does the model resonate with lived experience? Does it articulate the expressive grammar of Being?

This evaluative frame is consistent with Hartmut Rosa’s analysis of resonance as a mode of relational existence, in which adequacy is measured not by control or efficiency but by mutual affective attunement (Resonanz, p.285; (Rosa, 2016). Applied to ontology, such resonance testing enhances the Onto-Rhythmic Self by making certain that it remains structurally rigorous while remaining faithful to phenomenological disclosure.

### Genealogical and Methodological Deepening

Before articulating the implications of the Onto-Rhythmic Self, it is necessary to situate our proposal within a broader genealogy of selfhood and to clarify the methodological and phenomenological commitments that differentiate it from inherited paradigms. Our deepening features two purposes: it shows continuity with the long tradition of philosophical inquiry into subjectivity, and it highlights the necessity of departing from representational, synthetic, and computational accounts toward a model grounded in resonance, affect, and enactment.

#### Historical Genealogy of Selfhood

The trajectory of Western accounts of selfhood divulges a series of shifts and displacements. Hume’s “bundle theory” (Book I, Part IV, 6; (Hume, 2000) destabilized the notion of a substantial, persisting self by reducing it to a collection of perceptions, lacking intrinsic unity. Kant responded with the “transcendental unity of apperception” (A346/B404; (Kant, 1908), which reintroduced a unifying function, not empirical but structural, anticipating Husserl’s transcendental ego (Ideas I, 46–49). William James’s “stream of consciousness” (James, 1890) carried the perspective forward, offering a proto-phenomenological account of subjectivity as temporally flowing rather than statically given. Such stages frame Husserl’s phenomenology and Dennett’s functionalism as only two inflection points in a much longer genealogy—one that the Onto-Rhythmic Self quests to extend beyond both substantialist and reductionist frameworks.

#### Post-Structuralist Interventions

Where phenomenology sought eidetic grounding, post-structuralist thought introduced radical destabilization. Derrida’s analysis of différance (pp. 140–141; (Derrida, 1973) undermines Husserl’s (1913) attempt to ground temporal synthesis in absolute presence, showing that selfhood is always already displaced, deferred, and inscribed within spacing and delay. Foucault’s account of subjectivation (Hermeneutics of the Subject, 1982–83, pp. 369–372; (Gros, 2005) reframes the self as a historical and ethical practice—an ongoing labor of care, not a given essence. Taking together, such interventions signify the insufficiency of both transcendental and computational accounts: the self is neither an origin nor a fiction, but a practice of becoming within temporal and relational fields.

#### Methodological Expansion

Our hypotheses demand a methodology that can mediate between lived experience and scientific abstraction without collapsing in either. Neurophenomenology (Varela, 1996) provides such a bridge, combining first-person phenomenological reflection with third-person neuroscientific data. By contrast, contemporary computational models such as predictive processing and the free-energy principle (Friston, 2010; Hohwy, 2013) risk reducing subjectivity to optimization algorithms, extending Dennett’s narrative fiction into the domain of probabilistic coding. Such approaches sharpen our critique: where Husserl over-transcendentalizes (1913) and Dennett over-functionalizes (1993), the Onto-Rhythmic Self situates consciousness as resonance, an ontological event irreducible to either transcendental or computational structures.

#### Embodiment and Affectivity

To avoid abstraction, it is essential to foreground embodiment and affect. Merleau-Ponty’s account of the body as a pre-reflective “I can” (Merleau-Ponty, 1945) situates the self as an incarnate openness to the world, prior to reflection. Michel Henry’s theory of auto-affection (Michel, 1963) complements this by locating selfhood in affective self-experience rather than intentional representation. Such resources support our hypothesis that selfhood is rhythmic, enacted, and felt, not synthesized, or narrated.

#### Toward Future Implications

Positioned within such an expanded genealogy, the Onto-Rhythmic Self can be seen not as an isolated proposal but as a philosophical necessity. Its implications extend into clinical practice, where rhythmic engagement fosters temporal integration in trauma recovery and expressive therapies (Faulkner, 2017; McFerran et al., 2020). They also bear on debates in artificial intelligence, signifying that computation alone cannot generate resonance or affective depth, and thus cannot produce genuine selfhood. Finally, they open ethical vistas: if the self is a field of attunement rather than a substance or fiction, then moral responsibility lies in cultivating relational resonance—a perspective resonant with Levinas’s call to ethical responsibility before the Other (Levinas, 1961).

### Discussion: Reframing the Self as Ontic Modulation

The Onto-Rhythmic Self marks a paradigmatic shift in the understanding of subjectivity. Classical models have remained bound to the dualism of subject and object: either a constituting pole of experience, as in Husserl (1913), or a narrativized construct of symbolic processing, as in Dennett (1993). Our model dissolves such an opposition by articulating selfhood as ontic modulation—a semantic contour of Being enacted through resonance and temporal rhythm.

#### Husserl, Dennett, and the Limits of Subject or Simulation

For Husserl, the transcendental ego functions as the pole of intentional synthesis, grounding the constitution of objects in consciousness (Ideas I, 1913, 46–49). While such a framework provides a rigorous analysis of how meaning is constituted, it risks rendering selfhood a disembodied noetic center, insufficiently attentive to affective, embodied, and intersubjective resonance (Self-Awareness and Alterity, p.112; (Zahavi, 2005).

Dennett, by contrast, rejects transcendental idealism and posits the self as a “center of narrative gravity,” a fictional abstraction generated by language and cognitive modeling (Consciousness Explained, 1991, p.418). This functionalist move provides explanatory traction within cognitive science but reduces selfhood to symbolic simulation, thereby lacking existential depth (Mind in Life, p.19; (Thompson, 2010).

The Onto-Rhythmic Self intervenes precisely here: it refuses both the transcendental fiction of a constituting ego and the eliminativist fiction of a narrative center. Instead, it conceptualizes the self as a modulatory field of Being, where rhythm, meaning, and relational presence converge in ontological resonance.

#### Philosophical Reconfiguration: from Representation to Emergence

This reconceptualization aligns with what Deleuze and Derrida describe as differential ontology—where identity emerges from constitutive difference rather than stable essence (Deleuze, 1968; Difference and Repetition, p.28; Derrida, 1997; Of Grammatology, p.62; (Derrida, 1997). Individuation here is not a process of cognitive synthesis nor of narrative ordering, but of ontic emergence through resonance and temporal attunement.

Similarly, participatory epistemologies (The Passion of the Western Mind, p.433 (Tarnas, 2010); Revisioning Transpersonal Theory, p.121 (Ferrer & Tarnas, 2001) maintain that meaning arises through co-creative engagement with reality. The Onto-Rhythmic Self deepens this participatory turn, proposing that consciousness and time are not substrates but modalities of Being, enacted through rhythmic expression.

Our perspective also resonates with Hartmut Rosa’s sociological account of resonance, where self-world relations are not defined by control or representation but by mutual affective responsiveness (Resonanz, 2016, p.285). While Rosa develops such a notion in the context of modernity’s acceleration, the Onto-Rhythmic model ontologizes resonance itself, positioning it as the structural principle of subjectivity.

#### Clinical Implications: from Repair to Re-Attunement

Within clinical practice, models of selfhood often oscillate between cognitive-behavioral reconstruction and psychoanalytic narrative repair. Both presuppose a self as either system or story. By reframing the self as a modulatory field, the Onto-Rhythmic model shifts the clinical task from repair to re-attunement. Trauma, for example, can be understood not as a fracture in narrative coherence but as a dissonance in ontic rhythm. Our reframing parallels insights from music therapy, where rhythmic engagement has been shown to foster regulation, connection, and integration beyond discursive interventions (The Arts in Psychotherapy, 35:193–200 (Bensimon et al., 2008). The therapeutic task thus becomes restoring resonance rather than reconstructing representation.

#### Artistic Ontology: Creation as Ontic Inscription

Art, within representational paradigms, is often construed as symbolic depiction of inner states or external realities. Within the Onto-Rhythmic framework, however, artistic practice is understood as ontological inscription, a direct modulation of Being. Rhythm, gesture, and semantic layering do not solely represent but enact ontological presence. Such a move parallels Jan Zwicky’s notion of lyric philosophy, where philosophical expression occurs through resonance and intermodal coherence rather than linear propositional logic (p.47 (Zwicky, 1992). Artistic creation thus becomes an existential articulation rather than aesthetic ornament.

#### Conclusion: Listening to Being

Through the Onto-Rhythmic Self we re-explain subjectivity as a field of modulation, a rhythm of presence, a site of expressive unfolding. We reject the categories of processors, narrator, or transcendental architects. Instead, we posit the self as the way Being listens to itself.

Our work of intellect has signified three moves: first, diagnosing the ontological insufficiencies of Husserl’s transcendental synthesis (1913) and Dennett’s computational simulation (1993); second, articulating the Onto-Rhythmic Self as a participatory and resonant ontology; and third, proposing methodological avenues for evaluating and enacting such a model.

The “come outs” are not a new cognitive theory of mind but a new mode of listening—attuned to the semantic pulse of experience, the relational depth of presence, and the rhythmic grammar of Being. Correspondingly, the Onto-Rhythmic Self is not only a theoretical alternative but also an ontological necessity for rethinking subjectivity in the 21 st century.

## Data Availability

No datasets were generated or analysed during the current study.
